# Pararectal Epidermal Inclusion Cyst in a Pediatric Patient

**DOI:** 10.7759/cureus.60989

**Published:** 2024-05-24

**Authors:** Nour H Moosa, Hadeel Bozieh, Nermin Darawi, Fatima Hajjaj, Noor Awad, Firas Almasaid

**Affiliations:** 1 Faculty of Medicine, Palestine Polytechnic University, Hebron, PSE; 2 Pediatric Surgery, Governmental Hebron Hospital, Hebron, PSE

**Keywords:** gluteal region, gluteal area, fistula, epidermoid cyst, pararectal space, pelvic cavity, epidermal inclusion cyst

## Abstract

Epidermal inclusion cysts, commonly found cutaneously, rarely manifest in the pelvis. They are typically asymptomatic and often occur following trauma or surgical interventions. Imaging modalities, notably computed tomography (CT) scans and magnetic resonance imaging (MRI), play a crucial diagnostic role. Herein, we report a rare case of a four-year-old female with a complicated medical and surgical history, presented with pain in the right gluteal region in the setting of past history of abscess drainage in the same area. Imaging revealed a cystic lesion in the right pararectal space and a fistula extending between the pelvic cavity and gluteal region. A laparotomy was performed, and a histopathologic examination confirmed the diagnosis of an epidermal inclusion cyst with no evidence of malignancy.

## Introduction

Epidermal inclusion cysts, or epidermoid cysts, are cystic lesions that may develop in any part of the body, and they can be congenital or acquired [1]. Congenital lesions are caused by the misplacement of an ectodermal component during embryonic development. These cysts are common benign cutaneous lesions and are only found in the pelvis in around one in 40,000 to one in 63,000 with a higher incidence in women [2,3]. Acquired epidermoid cysts may form in areas that were previously exposed to trauma or surgery which led the epidermis to sequester in the dermis, allowing for gradual development of the lesion through core sloughing of dead cells [4]. They are frequently asymptomatic and may occur without genetic predisposition [5]. A pelvic computed tomography (CT) scan and magnetic resonance imaging (MRI) are the gold standards for diagnosis [1].

Herein, we present a case of a unique large pararectal epidermal inclusion cyst in a four-year-old female patient presenting with right gluteal pain. This research further highlights the important role of histopathology, as well as imaging modalities, in reaching a definitive diagnosis and the effective use of laparotomy inpatient treatment.

## Case presentation

A four-year-old female patient presented to the pediatric surgery outpatient clinic complaining of pain in the right gluteal region. The pain was localized and exacerbated by walking. The mother stated that pain was constant for 11 months after her child had an incision with drainage for a large abscess in the right gluteal area. Upon physical examination, the patient was irritable, her vitals were normal, and a rounded discolored skin depression was noticed in the right gluteal area with a small pus-draining opening in the center. The abdomen was soft and lax, and an abnormal gait was noticed.

Her past history was relevant for having a congenital solitary pelvic kidney on the left side, operated imperforate anus, and rectovestibular fistula. Also, she suffered from hydronephrosis twice, pelviureteric junction (PUJ) stenosis, and distal ureteric stenosis. Pyeloplasty, nephrostomy, and DJ stent placement were done on separate occasions.

**Figure 1:**
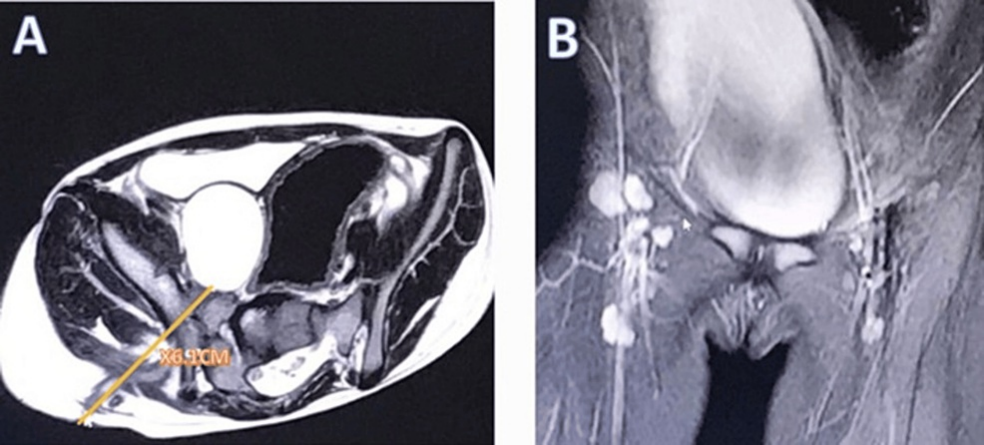
Magnetic resonance imaging (MRI): (A) Axial cross-section shows a large enhancing right gluteal collection with a deep fistula, the tract is about 6.1 cm long, indicating the described cystic lesion and both hips were normal; (B) the coronal cross-section shows the elongated cyst.

**Figure 2:**
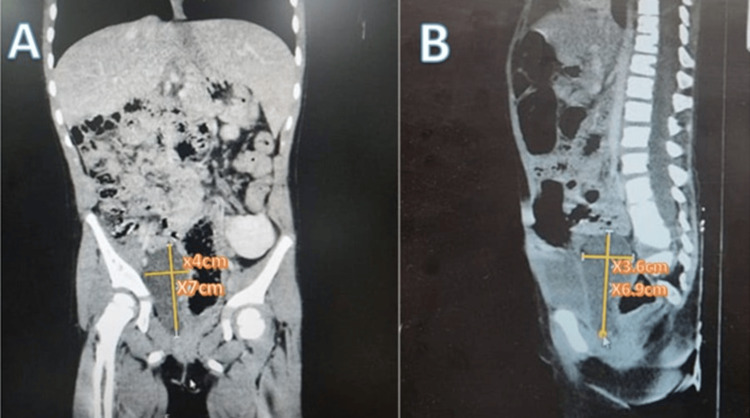
Computed tomography (CT). (A) Coronal view reveals a non-enhancing, well-defined cyst in the right pelvic cavity, measuring approximately 7 cm long and 4 cm wide. Additionally, a beaver tail liver was noted, along with mild scoliosis. No other significant issues were observed. (B) In the sagittal view, the depth of the cyst is measured as 3.6 cm, with the upper part located presacrally and the lower part posterior to the urinary bladder in the right pararectal space. No calcifications, septations, or soft tissue are present inside the cyst, and there is no wall enhancement.

The child was evaluated with ultrasound, MRI, and CT. Soft tissue ultrasound was done three months before the MRI, and the site of the lesion revealed a small collection measuring about 1.5 x 2 surrounded with edema and no right-side joint effusion. MRI was done two months before CT. Pelvic MRI without contrast revealed a large enhancing right gluteal collection measuring 4 cm x 3 cm, with a deep fistula indicating the pelvic cystic lesion (Figure 1). CT for the abdomen and pelvis with contrast showed a non-enhancing, well-defined cyst in the right pararectal space, elongated in shape, and measured 4 cm x 3.6 cm x 6.7 cm, suggesting a simple mesenteric cyst (Figure 2). The fistula connected the right gluteal region with the right pelvic cavity, measuring around 6.5 cm long. Contrast was injected into the fistula and passed from the surface of the skin to the pelvic cavity but not to the cystic lesion or urinary bladder.

**Figure 3:**
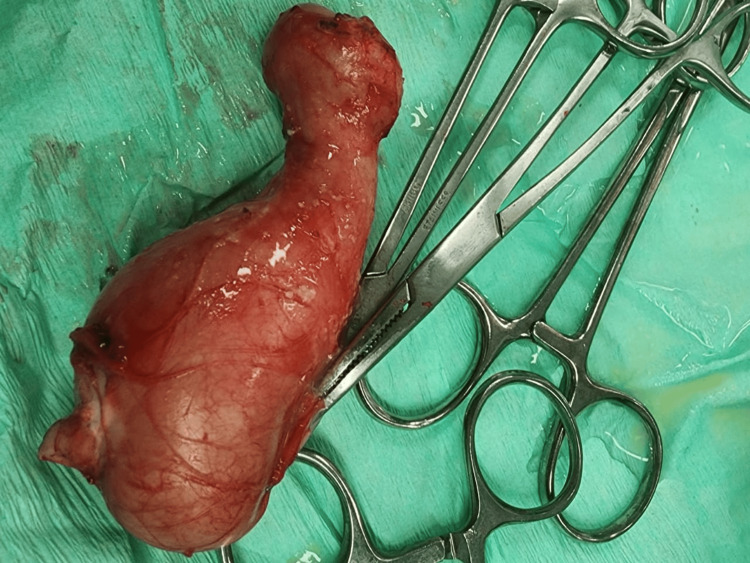
The excised cyst measuring 7 cm x 3.5 cm x 4 cm was removed carefully as one block; the wider part was presacral and the rest was pararectal.

Laparotomy under general anesthesia was done after one month to confirm diagnosis. Preoperatively, she had normal vitals, and all laboratory tests were within normal. Intraoperatively, methylene blue was injected into the fistula first, and then Pfannenstiel incision was done. The dye reached the pelvic cavity but not any structure or the cyst. Cyst was identified as an isolated structure and excised as one block (Figure 3). The histopathologic report revealed a cyst measuring 7 cm x 3.5 cm x 4 cm and filled with cheesy material. The cyst wall was made of dense fibrous stroma without adnexal features, lined by keratinizing squamous epithelium with a granular layer. The cyst lumen contained a large amount of laminated keratin material, confirming the diagnosis of an epidermal inclusion cyst with no evidence of malignancy.

Postoperative period course was uneventful. The patient was discharged on cefixime 250 mg and paracetamol syrup when necessary. Until two months after surgery, the patient did not attend the follow-up. We contacted the mother, and she reported that the child was still experiencing pain in the same area, and the fistula had not yet closed.

## Discussion

Pararectal epidermal inclusion cysts are congenital developmental cysts that originate from embryonic primordial germ layers in the hindgut. A congenital epidermoid cyst is unlikely in this case because the patient had several investigations previously, and it was not discovered earlier. Acquired cyst is more likely as a result of the procedures she had for her complicated condition. There are comparatively few research publications accessible on pararectal cystic lesions, which are uncommon phenomena. However, several investigations have been reported on retrorectal or presacral cystic lesions [6].

Usually slow-growing, pararectal epidermoid cysts are asymptomatic in 26% to 50% of patients. Urinary problems, constipation, perianal pain, or a palpable mass in the precoccygeal region are possible symptoms [7]. Infection, hemorrhage, or neoplastic degeneration are further complications [8]. It has been reported that 1% of epidermoid cysts will malignantly develop into basal cell carcinoma and squamous cell carcinoma [9]. Imaging procedures should be performed after a comprehensive physical examination and a detailed medical history [10].

An epidermoid cyst typically shows up on CT scans as a cystic mass with thin walls, fluid density, and maybe some calcification. Due to high protein content, past bleeding, or the deposition of pigments, including iron, it may exhibit major attenuation on a precontrast CT scan. The patterns of MR signals may vary. Cysts with a thin wall and heterogeneous signal intensity on T1- and T2-weighted MR images indicate an epidermoid cyst [6]. Misdiagnosis based on radiology is not unusual; case studies of this kind have documented developmental cysts like epidermoid, dermoid, rectal cystic duplication, and cystic hamartoma that have close imaging findings [11]. In the present case, CT and MRI suggested mesenteric dermoid cyst as the closest differential diagnosis, and the definitive diagnosis was only confirmed by the histopathology report.

Surgical excision of such lesions is required. Depending on the size, location, and relationship between the sacral vertebra and the lobulations, the method may change [8]. Cysts with a predominant intra-abdominal component frequently require an open or laparoscopic abdominal approach. Surgical resection of cystic tumors bordering the abdominal cavity that is situated in the upper rectum can be accomplished through an abdominal laparotomy. For big cysts extending above and beyond S3, a mixed abdominal-perineal method is recommended. For tumors below the mid-body of S3, posterior resection may be used. Other alternatives include a variety of procedures, such as trans-sacral or para-sacrococcygeal, posterior sagittal, intersphincteric, transsphincteric, or transsacrococcygeal approaches [12].

The finding of an epidermal inclusion cyst in the pelvis with this relatively large size in a pediatric female patient is very rare. To our knowledge, there have been only four prior cases reported in the literature, and this case is the first case in the Middle East [10,13-15]. Hence, it was discovered incidentally during the workup of the fistula observed during the physical examination, which was not connected to the cyst or the urinary bladder. Apart from that, this patient had a distinguished medical history since birth; she had a left single pelvic kidney and was born with an imperforated anus and rectovestibular fistula.

## Conclusions

While being regularly observed throughout the body, epidermoid cysts are rarely found in the pararectal space, particularly in a pediatric patient known for having a single left pelvic kidney. In this case, the site of the cyst was previously exposed to multiple surgical procedures. Various imaging techniques need to be employed to accurately diagnose and suggest appropriate treatment. Early diagnosis and appropriate treatment significantly reduce the risk of complications and recurrence. The mass should be completely removed, and histopathology should be done for confirmation. Providers need to maintain a wide differential diagnosis to identify the presence of a pelvic epidermoid cyst in children. This case illustrates how easy it is to misdiagnose a child with an epidermoid cyst due to a nonspecific presentation that overlaps with the remaining pain after the abscess drainage incision. More research is needed to determine the specific cause and progression of these cysts.
